# Uncertainty-Aware Visual Perception System for Outdoor Navigation of the Visually Challenged

**DOI:** 10.3390/s20082385

**Published:** 2020-04-22

**Authors:** George Dimas, Dimitris E. Diamantis, Panagiotis Kalozoumis, Dimitris K. Iakovidis

**Affiliations:** Department of Computer Science and Biomedical Informatics, University of Thessaly, 35131 Lamia, Greece; gdimas@uth.gr (G.D.); didiamantis@uth.gr (D.E.D.); pkalozoumis@uth.gr (P.K.)

**Keywords:** visually challenged, navigation, image analysis, fuzzy sets, machine learning

## Abstract

Every day, visually challenged people (VCP) face mobility restrictions and accessibility limitations. A short walk to a nearby destination, which for other individuals is taken for granted, becomes a challenge. To tackle this problem, we propose a novel visual perception system for outdoor navigation that can be evolved into an everyday visual aid for VCP. The proposed methodology is integrated in a wearable visual perception system (VPS). The proposed approach efficiently incorporates deep learning, object recognition models, along with an obstacle detection methodology based on human eye fixation prediction using Generative Adversarial Networks. An uncertainty-aware modeling of the obstacle risk assessment and spatial localization has been employed, following a fuzzy logic approach, for robust obstacle detection. The above combination can translate the position and the type of detected obstacles into descriptive linguistic expressions, allowing the users to easily understand their location in the environment and avoid them. The performance and capabilities of the proposed method are investigated in the context of safe navigation of VCP in outdoor environments of cultural interest through obstacle recognition and detection. Additionally, a comparison between the proposed system and relevant state-of-the-art systems for the safe navigation of VCP, focused on design and user-requirements satisfaction, is performed.

## 1. Introduction

According to the World Health Organization (WHO), about 16% of the worldwide population lives with some type of visual impairment [1]. Visually challenged people (VCP) struggle in their everyday life and have major difficulties in participating in sports, cultural, tourist, family, and other types of outdoor activities. The last two decades, a key solution to this problem has been the development of assistive devices able to help, at least partially, the VCP to adjust in the modern way of life and actively participate in different types of activities. Such assistive devices require the cooperation of researchers from different fields, such as medicine, smart electronics, computer science, and engineering. So far, as a result of this interdisciplinary cooperation, several designs and components of wearable camera-enabled systems for VCP have been proposed [2,3,4,5]. Such systems incorporate sensors, such as cameras, ultrasonic sensors, laser distance sensors, inertial measurement units, microphones, and GPS, which enable the user identify his/her position in an area of interest (i.e., outdoor environment, hospital, museum, archeological site, etc.), avoid static or moving obstacles and hazards in close proximity, and provide directions not only for navigation support but also for personalized guidance in that area. Moreover, mobile cloud-based applications [6], methodologies for optimal estimation of trajectories using GPS and other sensors accessible from a mobile device [7], and algorithms enabling efficient data coding for video streaming [8] can be considered for enhanced user experience in this context. Users should be able to easily interact with the system through speech in real-time. Moreover, the system should be able to share the navigation experience of the user not strictly as audiovisual information but also through social interaction with remote individuals, including people with locomotor disabilities and the elderly.

During the last two years, several studies and research projects have been initiated, setting higher standards for systems for computer-assisted navigation of VCP. In [9], an Enterprise Edition of a Google glass device was employed to support visually challenged individuals during their movement. Their system comprised a user interface, a computer network platform, and an electronic device to integrate all components into a single assistive device. In [10], a commercial pair of smart glasses (KR-VISION), consisting of an RGB-D sensor (RealSense R200) and a set of bone-conducting earphones, was linked to a portable processor. The RealSense R200 sensor was also employed in [11], together with a low-power millimeter wave (MMW) radar sensor, in order to unify object detection, recognition, and fusion. Another smart assistive navigation system comprised a smart-glass with a Raspberry Pi camera attached on a Raspberry Pi processor, as well as a smart shoe with an IR sensor for obstacle detection attached on an Arduino board [12]. In [13], a binocular vision probe with two charged coupled device (CCD) cameras and a semiconductor laser was employed to capture images in a fixed frequency. A composite head-mounted wearable system with a camera, ultrasonic sensor, IR sensor, button controller, and battery for image recognition was proposed in [14]. Two less complex approaches were proposed in [15,16]. In the first, two ultrasonic sensors, two vibrating motors, two transistors, and an Arduino Pro Mini Chip were attached on a simple pair of glasses. The directions were provided to the user through vibrations. In the second, a Raspberry Pi camera and two ultrasonic sensors attached on a Raspberry Pi processor were placed on a plexiglass frame.

Τhe aforementioned systems incorporate several types of sensors, which increase the computational demands and the energy consumption, the weight of the wearable device, as well as the complexity of the system. In addition, although directions in the form of vibrations are faster perceivable, their expressiveness is limited, and the learning curve required increases with the number of messages needed for user navigation. 

This paper presents a novel visual perception system (VPS) for outdoor navigation of the VCP in cultural areas, which copes with these issues in accordance with the respective user requirements [17]. The proposed system differs from others, because it follows a novel uncertainty-aware approach to obstacle detection, incorporating salient regions generated using a Generative Adversarial Network (GAN) trained to estimate saliency maps based on human eye-fixations. The estimated eye-fixation maps, expressing the human perception of saliency in the scene, adds to the intuition of the obstacle detection methodology. Additional novelties of the proposed VPS, include: (a) it can be personalized, based on the user characteristics—the user’s height, in order to minimize false alarms that may occur from the obstacle detection methodology and 3D printed to meet the user’s preferences; (b) the system implements both obstacle detection and recognition; and (c) the methodologies of obstacle detection and recognition are integrated in the system in a unified way.

The rest of this paper is organized in 6 sections, namely, Section 2, where the current state-of-the-art is presented; Section 3, describing the system architecture; Section 4, analyzing the methodologies used for obstacle detection and recognition; Section 5, examining the performance of the obstacle detection and recognition tasks; Section 6, where the performance of the proposed system with respect to other systems is discussed; and finally Section 7 presenting the conclusions of this work.

## 2. Related Work

### 2.1. Assistive Navigation Systems for the VCP

A review on relevant systems proposed until 2008 was presented in [18], where three categories of navigation systems were identified. The first category is based on positioning systems, including the Global Positioning System (GPS) for outdoor positioning, and preinstalled pilots and beacons emitting signals to determine the absolute position of the user in a local structured environment; the second is based on radio frequency identification (RFID) tags with contextual information, such as surrounding landmarks and turning points; and the third concerns vision-based systems that exploit information acquired from digital cameras to perceive the surrounding environment. Moreover, in a survey conducted in 2010 [19], wearable obstacle avoidance electronic travel aids for blind were reviewed and ranked based on their features. A more recent study [20] reviewed the state-of-the-art sensor-based assistive technologies, where it was concluded that most of the current solutions are still at a research stage, only partially solving the problem of either indoor or outdoor navigation. In addition, some guidelines for the development of relevant systems were suggested, including real-time performance (i.e., fast processing of the exchanged information between user and sensors and detection of suddenly appearing objects within a range of 0.5–5 m), wireless connectivity, reliability, simplicity, wearability, and low cost. 

In more recent vision-based systems, the main and most critical functionalities include the detection of obstacles, provision of navigational assistance, as well as recognition of objects or scenes in general. A wearable mobility aid solution based on embedded 3D vision was proposed in [21], which enables the user to perceive, be guided by audio messages and tactile feedback, receive information about the surrounding environment, and avoid obstacles along a path. Another relevant system was proposed in [4], where a stereo camera was used to perceive the environment, providing information to the user about obstacles and other objects in the form of intuitive acoustic feedback. A system for joint detection, tracking, and recognition of objects encountered during navigation in outdoor environments was proposed in [3]. In that system, the key principle was the alternation between tracking using motion information and prediction of the position of an object in time based on visual similarity. Another project [2] investigated the development of a smart-glass system consisting of a camera and ultrasonic sensors able to recognize obstacles ahead, and assess their distance in real-time. In [22], a wearable camera system was proposed, capable of identifying walkable spaces, planning a safe motion trajectory in space, recognizing and localizing certain types of objects, as well as providing haptic-feedback to the user through vibrations. A system named Sound of Vision was presented in [5], aiming to provide the users with a 3D representation of the surrounding environment, conveyed by means of hearing and tactile senses. The system comprised an RGB-depth (RGB-D) sensor and an inertial measurement unit (IMU) to track the head/camera orientation. A simple smart-phone-based guiding system was proposed in [23], which incorporated a fast feature recognition module running on a smart-phone for fast processing of visual data. In addition, it included two remotely accessible modules, one for more demanding feature recognition tasks and another for direction and distance estimation. In the context of assisted navigation, an indoor positioning framework was proposed by the authors of [24]. Their positioning framework is based on a panoramic visual odometry for the visually challenged people.

An augmented reality system using predefined markers to identify specific facilities, such as hallways, restrooms, staircases, and offices within indoor environments, was proposed in [25]. In [26], a scene perception system based on a multi-modal fusion-based framework for object detection and classification was proposed. The authors of [27] aimed to the development of a method integrated in a wearable device for the efficient place recognition using multimodal data. In [28], a unifying terrain awareness framework was proposed, extending the basic vision system based on an IR RGB-D sensor proposed in [10] and aiming at achieving efficient semantic understanding of the environment. The above approach, combined with a depth segmentation method, was integrated into a wearable navigation system. Another vision-based navigational aid using an RGB-D sensor was presented in [29], which solely focused on a specific component for road barrier recognition. Even more recently, a live object recognition blind aid system based on convolutional neural network was proposed in [30], which comprised a camera and a computer system. In [9], a system based on a Google Glass device was developed to navigate the user in unfamiliar healthcare environments, such as clinics, hospitals, and urgent cares. A wearable vision assistance system for visually challenged users based on big data and binocular vision sensors was proposed in [13]. Another assistive navigation system proposed in [12] combined two devices, a smart glass and a smart pair of shoes, where various sensors were integrated with Raspberry Pi, and the data from both devices are processed to provide more efficient navigation solutions. In [11], a low-power MMW radar and an RGB-D camera were used to unify obstacle detection, recognition, and fusion methods. The proposed system is not wearable but hangs from the neck of the user at the height of the chest. A navigation and object recognition system presented in [31] consisted of an RGB-D sensor and an IMU attached on a pair of glasses and a smartphone. A simple obstacle detection glass model, incorporating ultrasonic sensors, was proposed in [15]. Another wearable image recognition system, comprising a micro camera, an ultrasonic sensor, an infrared sensor, and a Raspberry Pi as the local processor, was presented in [14]. On the one side of the wearable device were the sensors and the controller and on the other the battery. In [32], a wearable system with three ultrasonic sensors and a camera was developed to recognize texts and detect obstacles and then relay the information to the user via an audio outlet device. A similar but less sophisticated system was presented in [16]. 

A relevant pre-commercial system, called EyeSynth (Audio-Visual System for the Blind Allowing Visually Impaired to See Through Hearing), promises both obstacle detection and audio-based user communication, and it is developed in the context of a H2020 funding scheme for small medium enterprises (SMEs). It consists of a stereoscopic imaging system mounted on a pair of eyeglasses, and non-verbal and abstract audio signals are communicated to the user. Relevant commercially available solutions include ORCAM MyEye, a device attachable to eyeglasses that discreetly reads printed and digital text aloud from various surfaces and recognizes faces, products, and money notes; eSight Eyewear, which uses a high-speed and high-definition camera that captures whatever the user sees and then displays it on two near-to-eye displays enhancing the vision of partially blind individuals; and the AIRA system, which connects blind or low-vision people with trained, remotely-located human agents who, at the touch of a button, can have access to what the user sees through a wearable camera. The above commercially available solutions do not yet incorporate any intelligent components for automated assistance. 

In the proposed system, barebone computer unit (BCU), namely a Raspberry Pi Zero, is employed, since it is easily accessible to everyone and easy to use, contrary to other devices such as Raspberry Pi processors. In contrast to haptic feedback or audio feedback in the form of short sound signals, the proposed method uses linguistic expressions incurring from fuzzy modeling to inform the user about obstacles, their position in space, and scene description. The human eye-fixation saliency used for obstacle detection provides the system with human-like eye-sight characteristics. The proposed method relies on visual cues provided only by a stereo camera system, instead of the various different sensors used in previous systems, thus reducing the computational demands, design complexity, and energy requirements, while enhancing user comfort. Furthermore, the system can be personalized according to the user’s height, and the wearable frame is 3D printed, therefore, adjusting to the preferences of each individual user, e.g., head anatomy, and avoiding restrictions imposed by using commercially available glass frames.

### 2.2. Obstacle Detection

Image-based obstacle detection is a component of major importance for assistive navigation systems for the VCP. A user requirement analysis [17], revealed that the users need a system that aims to real-time performance and mainly detects vertical objects, e.g., trees, humans, stairs, and ground anomalies.

Obstacle detection methodologies consists of two steps: (a) an object detection step and (b) an estimation step of the threat that an object poses to the agent/VCP. The image-based object detection problem has been previously tackled with the deployment of deep learning models. The authors of [33] proposed a Convolutional Neural Network (CNN) model, namely Faster Region-Based CNN, that was used for real-time object detection and tracking [26]. In [3], the authors proposed a joint object detection, tracking and recognition in the context of the DEEP-SEE framework. Regarding wearable navigation aids for VCP, an intelligent smart glass system, which exploits deep learning machine vision techniques and the Robotic Operating System, was proposed in [2]. The system uses three CNN models, namely, the Faster Region-Based CNN [33], You Only Look Once (YOLO) CNN model [34], and Single Shot multi-box Detectors (SSDs) [35]. Nevertheless, the goal of the aforementioned methods was solely to detect objects and not to classify them as obstacles.

In another work, a module of a wearable mobility aid was proposed based on the LeNet model for obstacle detection [21]. However, this machine learning method treats obstacle detection as a 2D problem. A multi-task deep learning model, which estimates the depth of a scene and extracts the obstacles without the need to compute a global map with an application in micro air vehicle flights, has been proposed in [35]. Other, mainly preliminary, studies have approached the obstacle detection problem for the safe navigation of VCP as a 3D problem by using images along with depth information and enhancing the performance by exploiting the capabilities of CNN models [36,37,38].

Aiming to robust obstacle detection, in this paper we propose a novel, uncertainty-aware personalized method, implemented by our VPS, based on a GAN and fuzzy sets. The GAN is used to detect salient regions within an image, where the detected salient regions are then combined with the 3D spatial information acquired by an RGB-D sensor using fuzzy sets theory. This way, unlike previous approaches, the proposed methodology is able to determine the level of threat posed by the obstacle to the user and its position in the environment with linguistic expressions. In addition, the proposed method takes into consideration the height of the user in order to describe the threat of an obstacle more efficiently. Finally, when compared to other deep learning assisted approaches, our methodology does not require any training regarding the obstacle detection part. 

### 2.3. Object Recognition

Although object detection has a critical role in the safety assurance of VCP, the VPS aims to provide an effective object and scene recognition module, which enables the user to make decisions based on the visual context of the environment. More specifically, object recognition provides the capability to the user to identify what type of object has been detected by the object detection module. Object recognition can be considered as a more complex module compared to object detection, since it requires an intelligent system that can incorporate the additional free parameters required to distinguish between the different detected objects. 

In the last decade, object recognition techniques have been drastically improved, mainly due to the appearance of CNN architectures, such as [39]. CNNs are a type of ANNs that consist of multiple convolutional layers with neuron arrangement mimicking the biological visual cortex. This enables CNNs to automatically extract features from the entire image, instead of relying on hand-crafted features, such as color and texture. Multiple CNN architectures have been proposed over the last years, each one contributing some unique characteristics [17]. Although conventional CNN architectures, such as the Visual Geometry Group Network (VGGNet) [40], offer great classification performance, they usually require large, high-end workstations equipped with Graphical Processing Units (GPUs) to execute them. This is mainly due to their large number of free-parameters [40] that increase their computational complexity and inference time, which in some applications, such as the assistance of VCP, is a problem of major importance. Recently, architectures, such as MobileNets [41] and ShuffleNets [42], have been specifically proposed to enable their execution on mobile and embedded devices. More specifically, MobileNets [41] are a series of architectures, which by using depth-wise separable convolutions [43] instead of conventional convolutions, vastly reduce the number of free-parameters of the network, enabling their execution on mobile devices. The authors in [42] proposed the use of the ShuffleNets architecture by using point-wise group convolution and channel shuffling to achieve a low number of free-parameters with high classification accuracy. Both architectures try to balance the trade-off between classification accuracy and computational complexity. 

CNNs have also been used for object and scene recognition tasks in the context of assisting VCP. In the work of [21], a mobility aid solution was proposed that uses a LeNet architecture for object categorization in 8 classes. An architecture named “KrNet” was proposed in [29], which relies on a CNN architecture to provide real-time road barrier recognition in the context of navigational assistance of VCP. A terrain awareness framework was proposed in [28] that uses CNN architectures, such as SegNet [44], to provide semantic image segmentation. 

In VPS, we make use of a state-of-the-art CNN architecture named Look Behind Fully Convolutional Network light or LB-FCN light [45], which offers high object recognition accuracy, while maintaining low computational complexity. Its architecture is based on the original LB-FCN architecture [46], which offers multi-scale feature extraction and shortcut connections that enhance the overall object recognition capabilities. LB-FCN light replaces the original convolutional layers with depth-wise separable convolutions and improves the overall architecture by extracting features under three different sizes (3 × 3, 5 × 5, and 7 × 7), lowering the number of free parameters of the original architecture. This enables the computationally efficient performance of the trained network while maintaining the recognition robustness, which is important for systems that require fast recognition responses, such as the one proposed in this paper. In addition to the low computational complexity provided by the LB-FCN light architecture, the system is cost-effective, since the obstacle recognition task does not require high-end expensive GPUs. Consequently, multiple conventional low-cost CPUs can be used instead, which enable relatively easy horizontal scaling of the system architecture. 

## 3. System Architecture

The architecture of the cultural navigation module of the proposed VPS, consists of four components; a stereoscopic depth-aware RGB camera, a BCU, a wearable Bluetooth speaker device, and cloud infrastructure. The first three components are mounted on a single smart wearable system, with the shape of sunglasses, capable of performing lightweight tasks, such as risk assessment, while the computationally intense tasks, such as object detection and recognition, are performed on a cloud computing infrastructure. These components are further analyzed in the following Section 3.1 and Section 3.2.

### 3.1. System Components and Infrastructure 

As the stereoscopic depth aware RGB camera, the Intel^®^ RealSense^TM^ D435 was chosen, since it provides all the functionalities needed by the proposed system in a single unit. This component is connected via a USB cable to a BCU of the wearable system. The BCU used in the system was a Raspberry Pi Zero. The BCU orchestrates the communication between the user and the external services that handle the computationally expensive deep learning requirements of the system on a remote cloud computing infrastructure. Another role of the BCU is to handle the linguistic interpretation of the detected objects in the scenery and communicate with the Bluetooth component of the system, which handles the playback operation. For the communication of the BCU component with the cloud computing component, we chose to use a low-end mobile phone that connects to the internet using 4G or Wi-Fi when available, effectively acting as a hotspot device.

For the communication between the BCU and the cloud computing component of the system, we chose to use the Hyper Text Transfer Protocol version 2.0 (HTTP/2), which provides a simple communication protocol. As the entry point of the cloud computing component, we used a load balancer HTTP microservice, which implements a REpresentational State Transfer (RESTful) Application Programming Interface (API) that handles the requests coming from the BCU, placing them in a message queue for processing. The queue follows the Advanced Message Queuing Protocol (AMQP), which enables a platform agnostic message distribution. A set of message consumers, equipped with Graphical Processing Units (GPUs), process the messages that are placed in the queue and, based on the result, communicate back to the MPUs using the HTTP protocol. This architecture enables the system to be extensible both in terms of infrastructure, since new works can be added on demand, and in terms of functionality, depending on future needs of the platform. 

**Figure 1:**
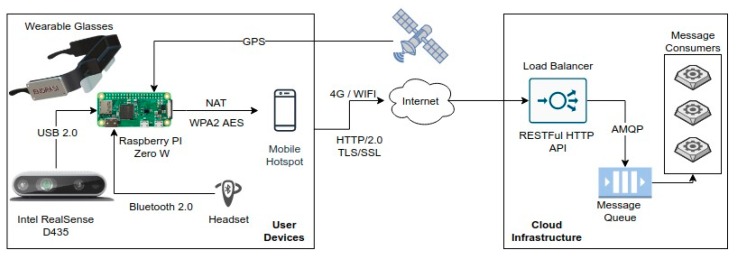
Visual perception system (VPS) architecture overview illustrating the components of the system along with their interconnectivity.

The VPS component communication is shown in Figure 1. More specifically, the BCU component of the system, receives RGB-D images from the stereoscopic camera at a real-time interval. Each image is then analyzed using fuzzy logic by the object detection component of the system on the BCU itself, performing risk assessment. In parallel, the BCU communicates with the cloud computing component by sending a binary representation of the image to the load balancer, using the VPS RESTful API. A worker then receives the message placed in the queue from the load balancer and performs the object detection task, which involves the computation of the image saliency map from the received images using a GAN. When an object is detected and its boundaries determined, the worker performs the object recognition task using a CNN, the result of which is a class label for each detected object in the image. The worker, using HTTP, informs the MPU about the presence and location of the object in the image along with the detected labels. As a last step, the MPU linguistically translates the object position along with the detected labels provided from the methodology described in Section 4, using the build-in text to speech synthesizer of the BCU. The result is communicated via Bluetooth with the speaker attached to the ear of the user for playback. It is important to mention here that, in case of repeated object detections, the BCU component avoids the playback of the same detected object based on the change of the scenery, which enables the system to prevent unnecessary playbacks. In detail, as users are approaching an obstacle, the system notifies them about the collision risk, which is described using the linguistic expressions low, medium and high and its spatial location and category. To avoid user confusion, the system implements a controlled notification policy, where the frequency of notifications increases as the users are getting closer to the obstacle. The information about the obstacle’s spatial location and category are provided only in the first notification of the system. If the users continue moving towards a high-risk obstacle, the system notifies them with a “stop” message.

### 3.2. Smart Glasses Design

The wearable device, in the form of smart glasses, was designed using a CAD software according to the user requirements listed in [17]. The most relevant to the design requirements mentioned that the wearable system should be attractive and elegant, possibly with a selection of different colors, but in a minimalist rather than attention grabbing way. In terms of construction, the system should be robust; last a long time, not requiring maintenance; and be resistant to damage, pressure, knocks and bumps, water, and harsh weather conditions [17]. 

**Figure 2:**
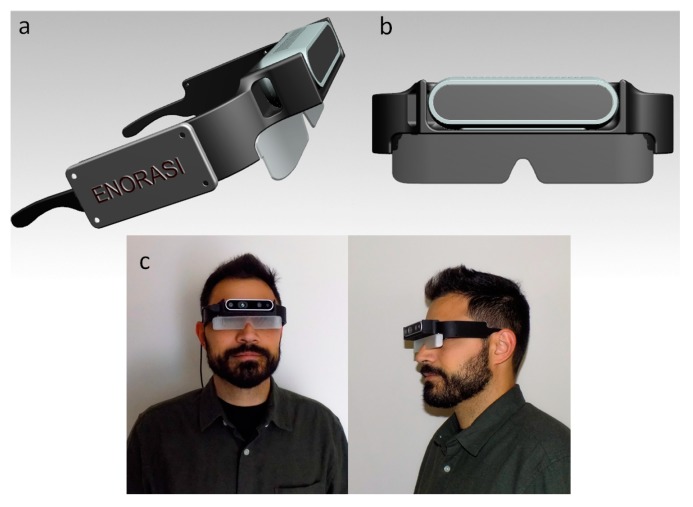
3D representation of the smart glasses. (**a**) Side view of the glasses; (**b**) front view of the glasses; and (**c**) 3D-printed result with the actual camera sensor. In this preliminary model, the glass-part was printed with transparent PLA filament, which produced a blurry, semi-transparent result. In future versions, the glass-part will be replaced by transparent polymer or glass.

The design of the model has been parameterized, in terms of its width and length, making it highly adjustable. Therefore, it can be easily customized for each user based on the head dimensions, which makes it more comfortable. The model (Figure 2a,b) comprises two parts, the frame and the glass. In the front portion of the frame, there is a specially designed socket, where the Intel^®^ RealSense^TM^ D435 camera can be placed and secured with a screw at its bottom. In addition, the frame has been designed to incorporate additional equipment if needed, such as Raspberry Pi (covered by the lid with the VPS logo), an ultrasonic sensor, and an IMU. The designed smart-glass model was 3D printed using PLA filament in a Creality CR-10 3D printer. The resulted device is illustrated in Figure 2c.

## 4. Obstacle Detection and Recognition Component

**Figure 3:**
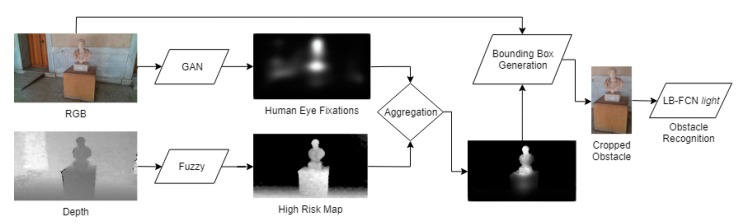
Visualization of the proposed obstacle detection and recognition pipeline.

The obstacle detection and recognition component can be described as a two-step process. In the first step, the detection function incorporates a deep learning model and a risk assessment approach using fuzzy sets. The deep learning model is used to predict, eye-human fixations, on images captured during the navigation of the VCP. Then, fuzzy sets are used to assess the risk based on depth values calculated by the RGB-D camera, generating risk maps, expressing different degrees of risk. The risk and saliency maps are then combined using a fuzzy aggregation process through which the probable obstacles are detected. In the second step, the recognition of the probable obstacles takes place. For this purpose, each obstacle region is propagated to a deep learning model, which is trained to infer class labels for objects found in the navigation scenery (Figure 3).

### 4.1. Obstacle Detection

The detection-recognition methodology can be summarized as follows:

(a) Eye human fixation estimation model;
(b) Depth-aware fuzzy risk assessment in the form of risk maps;
(c) Obstacle detection and localization via the fuzzy aggregation of saliency maps, produced in Step (a) and the risk maps produced in Step (b);
(d) Obstacle recognition using a deep learning model based on probable obstacle regions obtained in Step (c).

#### 4.1.1. Human Eye Fixation Estimation

**Figure 4:**
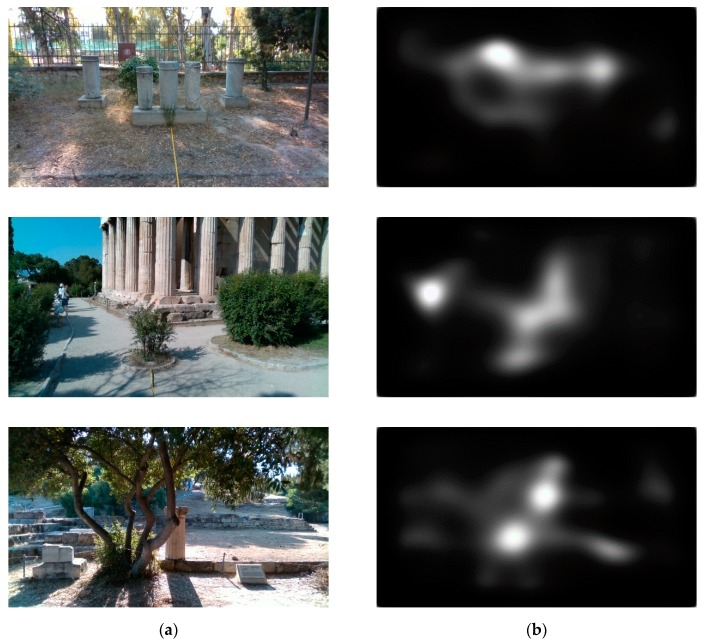
Examples of the generated saliency maps given an RGB image. (**a**) Input RGB images. (**b**) Respective generated saliency maps.

The saliency maps used in this work are generated by a GAN [47]. The generated saliency maps derive from human eye fixation points and thus, they make the significance of a region in a scene more instinctual. Such information can be exploited for the obstacle detection procedure, and at the same time, enhance the intuition of the methodology. Additionally, the machine learning aspect enables the extensibility of the methodology, since it can be trained with additional eye fixation data, collected from individuals during their navigation through rough terrains. An example of the saliency maps estimated from a given image can be seen in Figure 4. Since the model is trained on human eye-fixation data, it identifies as salient those regions in the image on which the attention of a human would be focused. As it can be observed in Figure 4, in the first image, the most salient region corresponds to the fire extinguisher cabinet; in the second image, to the people on the left side; and in the last image, to the elevated ground and the tree branch.

The GAN training utilizes two different CNN models, namely, a discriminator and a generator. During the training, the generator learns to generate imagery related to a task, and the discriminator assists to the optimization of the resemblance to the target images. In our case, the target data are composed of visual saliency maps based on human eye tracking data. 

The generator architecture is a VGG-16 [40] encoder-decoder model. The encoder follows an identical architecture to that of VGG-16 unaccompanied by fully connected layers. The encoder is used to create a latent representation of the input image. The encoder weights are initialized by training the model on the ImageNet dataset [48]. During the training, there was no update of the weights of the encoder, with an exception to the last two convolutional blocks.

The decoder has the same architectural structure with the encoder network, with the exception that the layers are placed in reverse order, and the max pooling layers are replaced with up-sampling layers. To generate the saliency map, the decoder has an additional 1 × 1 convolutional layer in the output, with sigmoidal activation. The decoder weights were initialized randomly. The generator accepts an RGB image *I_RGB_* as stimulus and generates a saliency map that resembles the human eye fixation on that *I_RGB_*.

**Figure 5:**
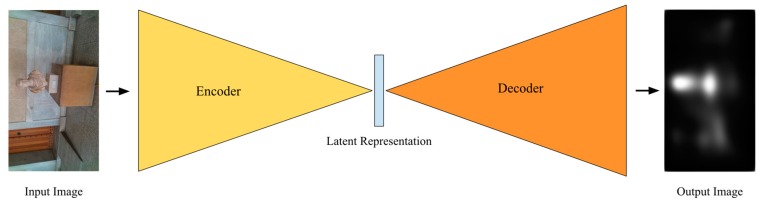
Illustration of the generator architecture. The generator takes as input an RGB image *I_RGB_* and outputs a saliency map based on human eye fixation.

The discriminator of the GAN has a simpler architecture. The discriminator model consists of 3 × 3 convolutional layers, combined with 3 max pooling layers followed by 3 Fully Connected (FC) layers. The Rectified Liner Unit (ReLU) and hyperbolic tangent (tanh) functions are deployed as activation functions for the convolutional and FC layers, respectively. The only exception is the last layer of the FC part, where the sigmoid activation function was used. The architecture of the GAN generator network is illustrated in Figure 5.

#### 4.1.2. Uncertainty-Aware Obstacle Detection

In general, an object that interferes with the safe navigation of a person can be perceived as salient. Considering this, the location of an obstacle is likely to be in regions of a saliency map that indicate high importance, i.e., with high intensities. A saliency map produced by the model described in Section 4.1.1 can be treated as a weighted region of interest, in which an obstacle may be located. High-intensity regions of such a saliency map indicate high probability of the presence of an object of interest. Among all the salient regions in the saliency map, we need to identify these regions that may pose a threat to the person navigating in the scenery depicted in *I_RGB_*. Thus, we follow an approach, where both a saliency map and a depth map deriving by an RGB-D sensor are used for the risk assessment. The combination of the saliency and depth maps is achieved with the utilization of Fuzzy Sets [49].

**Figure 6:**
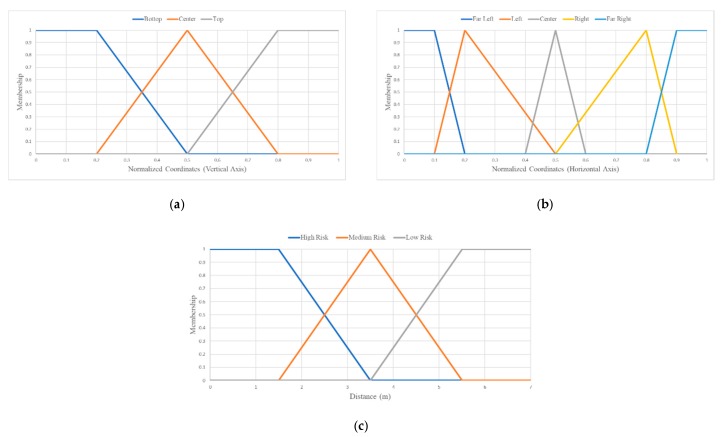
Membership functions of fuzzy sets used for the localization of objects in the 3D space using linguistic variables. (**a**) Membership functions for far left (*h*_1_), left (*h*_2_), central (*h*_3_), right (*h*_4_) and far right (*h*_5_) positions on the horizontal axis. (**b**) Membership functions for up (*v*_1_), central (*v*_2_) and bottom (*v*_3_) positions on the vertical axis. (**c**) Membership functions for low (*r*_1_), medium (*r*_1_), and high risk (*r*_3_) upon the distance of the user from an obstacle.

For assessing the risk, it can be easily deduced that objects/areas that are close to the VCP navigating in an area and are salient with regard to the human gaze may pose a certain degree of threat to the VCP. Therefore, as a first step, the regions that are in a certain range from the navigating person need to be extracted, so that they can be determined as threatening. Hence, we consider a set of 3 fuzzy sets, namely, *R*_1_, *R*_2_, and *R*_3_—describing three different risk levels, which can be described with the linguistic values of high, medium, and low risk, respectively. The fuzzy sets *R*_1_, *R*_2_, and *R*_3_ represent a different degree of risk and their universe of discourse is the range of depth values of a depth map. Regarding the fuzzy aspect of these sets and taking into consideration the uncertainty in the risk assessment, there is an overlap between the fuzzy sets describing low and medium and medium and *high* risk. The fuzzy sets *R*_1_, *R*_2_, and *R*_3_ are described by the membership function *r_i_(z), i =* 1, 2, 3, where *z* ∈ [0, ∞). The membership functions are illustrated in Figure 6c.

A major aspect of an obstacle detection methodology is the localization of obstacles and the description of their position in a manner that can be communicated and easily perceived by the user. In our system, the description of the spatial location of an object is performed using linguistic expressions. We propose an approach based on fuzzy logic to interpret the obstacle position using linguistic expressions (linguistic values) represented by fuzzy sets. Spatial localization of an obstacle in an image can be achieved by defining 8 additional fuzzy sets. More specifically, we define 5 fuzzy sets for the localization along the horizontal axis of the image, namely, *H*_1_, *H*_2_*, H*_3_, *H*_4_, and *H*_5_ corresponding to far left, left, central, right, and far right portions of the image. Additionally, to express the location of the obstacle along the vertical axis of the image, we define 3 fuzzy sets, namely, *V*_1_, *V*_2_, and *V*_3_ denoting the upper, central, and bottom portions of the image. The respective membership functions of these fuzzy sets are *h_j_(x)*, *j =* 1, 2, 3, 4, 5 and *v_i_(y), i =* 1, 2, 3, where *x, y* ∈ [0, 1] are normalized image coordinates. An illustration of these membership functions can be seen in Figure 6.

Some obstacles, such as tree branches, may be in close proximity to the individual with respect to the depth but at a certain height that safe passage would not be affected. Thus, a personalization step was introduced to the methodology eliminating false alarms. The personalization aspect and the minimization of false positive obstacle detection instances are implemented through an additional fuzzy set *P*, addressing the risk an obstacle poses to a person with respect to the height. For the description of this *P* fuzzy set, we define a two dimensional membership function *p*(*h_o_, h_u_*), where *h*_o_ and *h*_u_ are the heights of the obstacle and the user, respectively. The personalization methodology is described in Section 4.1.3.

**Figure 7:**
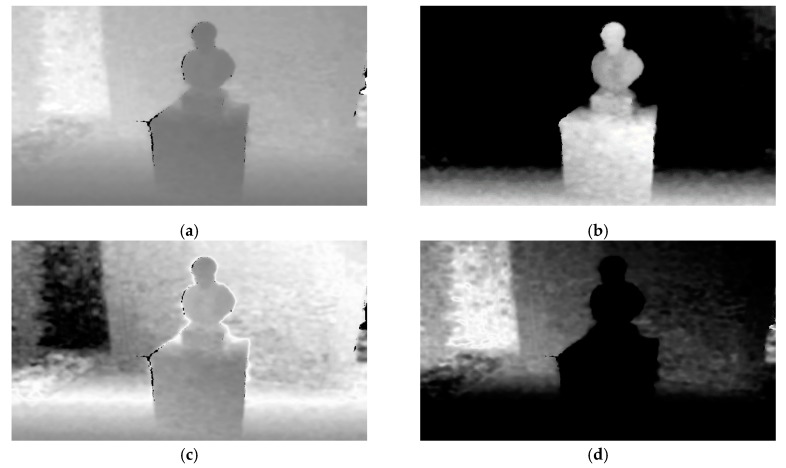
Example of $R_{M}^{i}$ creation. (**a**) Depth map *D,* where lower intensities correspond to closer distances; (**b**) visual representation of $R_{M}^{1}$ representing regions of high risk; (**c**) $R_{M}^{2}$ representing regions of medium risk; (**d**) $R_{M}^{3}$ depicting regions of low risk. Higher intensities in (**b–d**) correspond to lower participation in the respective fuzzy set. All images have been normalized for better visualization.

For the risk assessment, since the membership functions describing each fuzzy set were defined, the next step is the creation of 3 risk maps, $R_{M}^{i}$. The risk maps $R_{M}^{i}$, derive from the responses of a membership function, *r_i_(z)*, and are formally expressed as:

(1) $$R_{M}^{i}\left(x,\;y\right)=r_{i}\left(D\left(x,y\right)\right)$$

where *D* is a depth map that corresponds to an RGB image *I_RGB_*. Using all the risk assessment membership functions, namely *r*_1_, *r*_2_, and *r*_3_, 3 different risk maps, $R_{M}^{1}$, $R_{M}^{2}$, and $R_{M}^{3}$, are derived. Each of these risk maps depicts regions that may pose different degrees of risk to the VCP navigating in the area. In detail, risk map $R_{M}^{1}$ represents regions that may pose high degree of risk, $R_{M}^{2}$ medium degree of risk, and finally $R_{M}^{3}$ low degree of risk. A visual representation of these maps can be seen in Figure 7. Figure 7b,c illustrates the risk maps derived from the responses of the *r*_1_*, r*_2_, and *r*_3_ membership functions on the depth map of Figure 7a. Brighter pixel intensities represent higher participation in the respective fuzzy set, while darker pixel intensities represent lower participation.

In the proposed methodology, the obstacle detection is a combination between the risk assessed from the depth maps and the degree of saliency that is obtained from the GAN described in the previous subsection. The saliency map *S_M_* that is produced from a given *I_RGB_* is aggregated with each risk map $R_{M}^{i}$, where *i =* 1, 2, 3, using the fuzzy AND (∧) operator (Godel t-norm) [50], formally expressed as:

(2) $$F_{1}\wedge F_{2}=\min\left(F_{1}\left(x,\;y\right),F_{2}\left(x,\;y\right)\right)$$

In Equation (2), *F*_1_ and *F*_2_ denote two generic 2D fuzzy maps with values within the [0, 1] interval, and *x*, *y* are the coordinates of each value of the 2D fuzzy map. The risk maps $R_{M}^{i}$ are, by definition, fuzzy 2D maps, since they derive from the responses of membership functions *r_i_* on a depth map. The saliency map *S_M_* can be considered as a fuzzy map where its values represent the degree of participation of a given pixel to the salient domain. Therefore, they can be combined with the fuzzy AND operator to produce a new fuzzy 2D map $O_{M}^{i}$ as follows:

(3) $$O_{M}^{i}=R_{M}^{i}\wedge S_{M}$$

**Figure 8:**
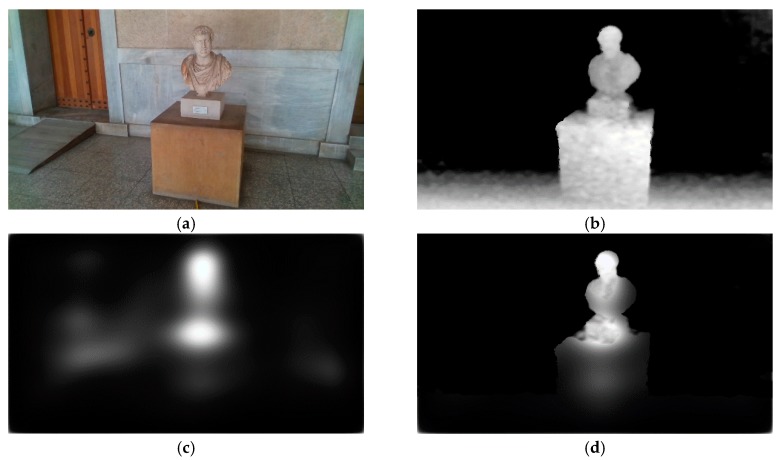
Example of the aggregation process between the saliency map SM and the high-risk map $R_{M}^{1}$. (**a**) Original I_RGB_ used for the generation of the saliency map SM; (**b**) high-risk map $R_{M}^{1}$ used in the aggregation; (**c**) saliency map S_M_ based on the human eye fixation on image (**a**); (**d**) the aggregation product using the fuzzy AND operator between images (**b**) and (**c**).

The non-zero values of the 2D fuzzy map $O_{M}^{i}$ (obstacle map) at each coordinate (*x, y*) indicate the location of an obstacle and express the degree of participation in the risk domain of the respective $R_{M}^{i}$. Figure 8d illustrates the respective $O_{M}^{i}$ produced using the fuzzy AND operator with the three $R_{M}^{i}$. Higher pixel values of the $O_{M}^{i}$ portray higher participation on the respective risk category and the probability of the location of an obstacle.

Theoretically, the $O_{M}^{i}$ can be directly used to detect obstacles posing different degrees of risk to the VCP navigating in the area. However, if the orientation of the camera is towards the ground, the ground plane can be often falsely perceived as obstacle. Consequently, a refinement step is needed to optimize the obstacle detection results and reduce the occurrence of false alarm error. Therefore, a simple but effective approach for ground plane extraction is adopted.

The ground plane has a distinctive gradient representation along the *Y* axis in depth maps, which can be exploited in order to remove it from the $O_{M}^{i}$. As a first step, the gradient of the depth map *D* is estimated by:

(4) $$\nabla D=\left(\frac{\partial D}{\partial x},\;\frac{\partial D}{\partial y}\right)$$

**Figure 9:**
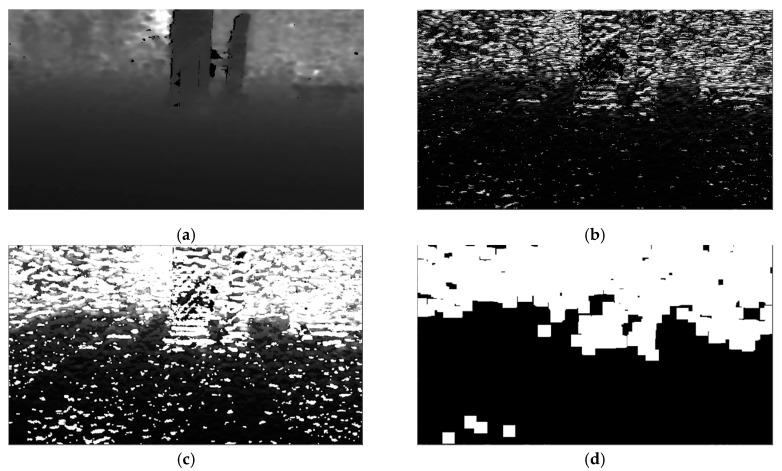
Example of the creation steps of *G_M_*. (**a**) Depth map *D*, normalized for better visualization; (**b**) visual representation of the difference map *Δ_M_*; (**c**) difference map *Δ_M_* after the application of the basic morphological gradient; and (**d**) the final ground removal mask *G_M_*.

**Figure 10:**
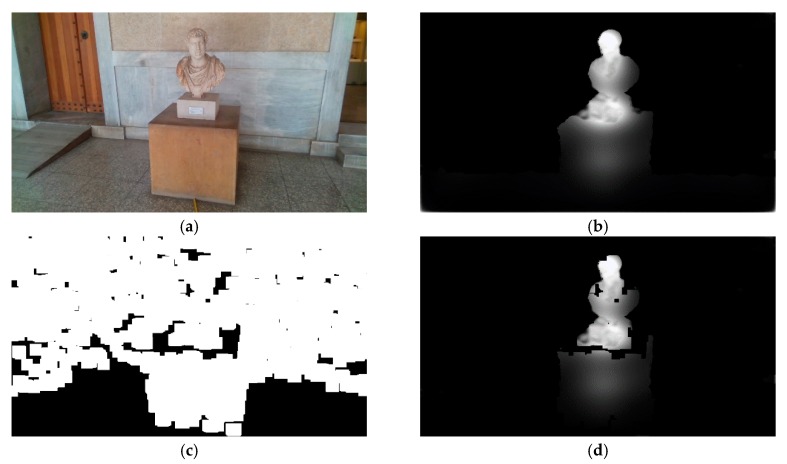
Example of the ground removal procedure. (**a**) Original *I_RGB_* image; (**b**) corresponding obstacle map $O_{M}^{1}$; (**c**) respective ground removal mask G_M_; (**d**) masked obstacle map $O_{M}^{1}$. In (**d**), the ground has been effectively removed.

A visual representation of a normalized difference map $\frac{\partial D}{\partial y}$ in the [0, 255] interval can be seen in Figure 9. As it can be seen, the regions corresponding to the ground have smaller differences than the rest of the depth map. In the next step, a basic morphological gradient *g* [51] is applied on the gradient of *D* along the *y* direction $\frac{\partial D}{\partial y}$. A basic morphological gradient is basically the difference between dilation and erosion of the $\frac{\partial D}{\partial y}$ given an all-one kernel *k*_5×5_:

(5) $$g\left(\frac{\partial D}{\partial y}\right)=\delta_{k_{5\times 5}}\left(\frac{\partial D}{\partial y}\right)-\varepsilon_{k_{5\times 5}}\left(\frac{\partial D}{\partial y}\right)$$

where *δ* and *ε* denote the operations of dilation and erosion and their subscripts indicate the used kernel. In contrast to the usual gradient of an image, the basic morphological gradient *g* corresponds to the maximum variation in an elementary neighborhood rather than a local slope. The morphological gradient is followed by consecutive operations of erosion and dilation with a kernel *k_5×5_*. As it can be noticed in Figure 9c, the basic morphological filter *g* gives higher responses on non-ground regions, and thus, the following operations of erosion and dilution are able to eliminate the ground regions quite effectively. The product of these consecutive operations is a ground removal mask *G_M_*, which is then multiplied with $O_{M}^{i}$, setting the values corresponding to the ground, to zero. This ground removal approach has been experimentally proven to be sufficient (Section 5) to eliminate the false identification of the ground as obstacle. A visual representation of the ground mask creation and the ground removal can be seen in Figure 9 and Figure 10, respectively.

Once the obstacle map of the depicted scene is estimated following the process described above, the next step is the spatial localization of the obstacle in linguistic values. This step is crucial for the communication of the surroundings to a VCP. For this purpose, Fuzzy Sets are utilized in this work. As presented in Section 4.1.1, 5 membership functions are used to determine the location of an obstacle along the horizontal axis (*x*-axis) and 3 along the vertical axis (*y*-axis).

**Figure 11:**
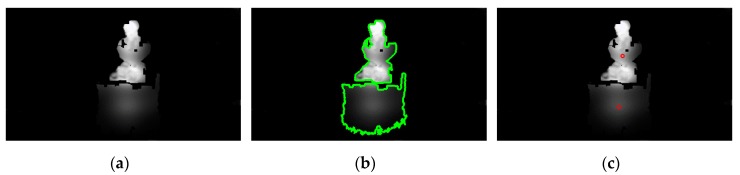
Example of the obstacle boundary extraction and obstacle center calculation. (**a**) $O_{P}^{1}$ obstacle map used for the detection of high-risk obstacles; (**b**) boundary (green outline) estimation of the obstacles; (**c**) respective centers of the detected obstacles.

Initially, the boundaries of the obstacles depicted in the obstacle maps need to be determined. For the obstacle detection task, the $O_{M}^{1}$ obstacle map, through which the high-risk obstacles are represented, is chosen. Then, the boundaries *b_l_*, where *l* = 1, 2, 3…, of the obstacles are calculated using a border following the methodology presented in [52]. Once the boundaries of each probable obstacle depicted in $O_{M}^{1}$ are acquired, their centers *c_l_* = (*c_x_*, *c_y_*), *l* = 1, 2, 3, … are derived by exploiting the properties of the image moments [53] of boundaries *b_l_*. The centers *c_l_* can be defined using the raw moments *m*_00_, *m*_10_, and *m*_01_ of *b_l_* as follows:

(6) $$m_{qk}=\iint_{b_{l}}x^{q}y^{k}I_{RGB}\left(x,\;y\right)dxdy$$

(7) $$c_{l}=\left(\frac{m_{10}}{m_{00}},\;\frac{m_{01}}{m_{00}}\right)$$

where *q* = 0, 1, 2, …, *k* = 0, 1, 2, … and *x, y* denote image coordinates along the *x*-axis and *y*-axis respectively. An example of the obstacle boundary detection can be seen in Figure 11, where the boundaries of the obstacles are illustrated with green lines (Figure 11b) and the centers of the obstacles are marked with red circles (Figure 11c).

Once the centers have been calculated, their location can be determined and described with linguistic values using the horizontal and vertical membership functions, *h_j_*, where *j* = 1, 2, 3, 4, 5, and *v_i_*, where *i* = 1, 2, 3. If the response of *h_j_*(c_x_) and *v_i_*(*c_y_*) is greater than 0.65, then the respective obstacle with a boundary center of *c_l_* = (*c_x_, c_y_*) will be described with the linguistic value that these *h_j_* and *v_i_* represent. Additionally, the distance between object and person is estimated using the depth value of depth map *D* at the location of *D*(*c_x_, c_y_*). Using this information, the VCP can be warned regarding the location and distance of the obstacle and, as an extension, be assisted to avoid it.

#### 4.1.3. Personalized Obstacle Detection Refinement

The obstacle map depicts probable obstacles that are salient for humans and are within a certain range. However, this can lead to false positive indications, since some obstacles, such as tree branches, can be within a range that can be considered threatening, but at a height greater than that of the user, not affecting his/her navigation. False positive indications of this nature can be avoided using the membership function *p*(*h_o_, h_u_*). To use this membership function, the 3D points of the scene need to be determined by exploiting the intrinsic parameters of the camera and the provided depth map.

To project 2D points on the 3D space in the metric system (meters), we need to know the corresponding depth value *z* for each 2D point. Based on the pinhole model, which describes the geometric properties of our camera [54], the projection of a 3D point to the 2D image plane is described as follows:

(8) $$\left(\begin{array}{c} \tilde{u}\\ \tilde{v} \end{array}\right)=\frac{f}{z}\left(\begin{array}{c} X\\ Y \end{array}\right)$$

where *f* is the effective focal length of camera, and (*X*, *Y, z*)^T^ is the 3D point corresponding to a 2D point on the image plane ${\left(\tilde{u},\;\tilde{v}\right)}^{T}$. Once the projected point ${\left(\tilde{u},\;\tilde{v}\right)}^{T}$ is acquired, the transition to pixel coordinates (*x, y*)^T^ is described by the following equation:

(9) $$\left(\begin{array}{c} x\\ y \end{array}\right)=\left(\begin{array}{c} D_{u}s_{u}\tilde{u}\\ D_{v}\tilde{v} \end{array}\right)+\left(\begin{array}{c} x_{0}\\ y_{0} \end{array}\right)$$

*s_u_* denotes a scale factor; *D_u_*, *D_v_* are coefficients needed for the transition from the metric units to pixels, and (*x_0_, y_0_*)^T^ is the principal point of the camera. With the combination of Equations (8) and (9) the projection which describes the transition from 3D space to the 2D image pixel coordinate system can be expressed as

(10) $$\left(\begin{array}{c} x\\ y \end{array}\right)=\left(\begin{array}{c} \frac{fD_{u}s_{u}X}{z}\\ \frac{fD_{v}Y}{z} \end{array}\right)+\left(\begin{array}{c} x_{0}\\ y_{0} \end{array}\right)$$

The 3D projection of a 2D point with pixel coordinates (*x, y*), for which the depth value *z* is known, can be performed by solving Equation (10) for *X*, *Y* formally expressed below [55]:

(11) $$\left(\begin{array}{c} X\\ Y \end{array}\right)=z\left(\begin{array}{c} \frac{x-x_{0}}{f_{x}}\\ \frac{y-y_{0}}{f_{y}} \end{array}\right)$$

where *f_x_* = *fD_u_s_u_* and *fy* = *fD_v_*. Equation (11) is applied on all the 2D points of *I_RGB_* with known depth values *z*. After the 3D points have been calculated, the *Y* coordinates are used to create a 2D height map *H*_M_ of the scene, where each value is a *Y* coordinate indicating the height an object at the corresponding pixel coordinate in *I_RBG_*. Given the height *h_u_* of the user, we apply the *p* membership function on the height map *H_M_* to assess the risk with respect to the height of the user. The responses of *p* on *H_M_* create a 2D fuzzy map *P_M_* as shown below:

(12) $$P_{M}\left(x,\;y\right)=p\left(H_{M}\left(x,y\right),\;hu\right)$$

Finally, the fuzzy AND operator is used to combine $O_{M}^{i}$ with P_M_, resulting in a final personalized obstacle map $O_{P}^{i}$:

(13) $$O_{P}^{i}=O_{M}^{i}\wedge P_{M}$$

Non-zero values of $O_{P}^{i}$ represent the final location of a probable obstacle with respect to the height of the user and the degree of participation to the respective risk degree, i.e., the fuzzy AND operation between $O_{P}^{1}$ with *P_M_* describes the high-risk obstacles in the scenery.

### 4.2. Obstacle Recognition

**Figure 12:**
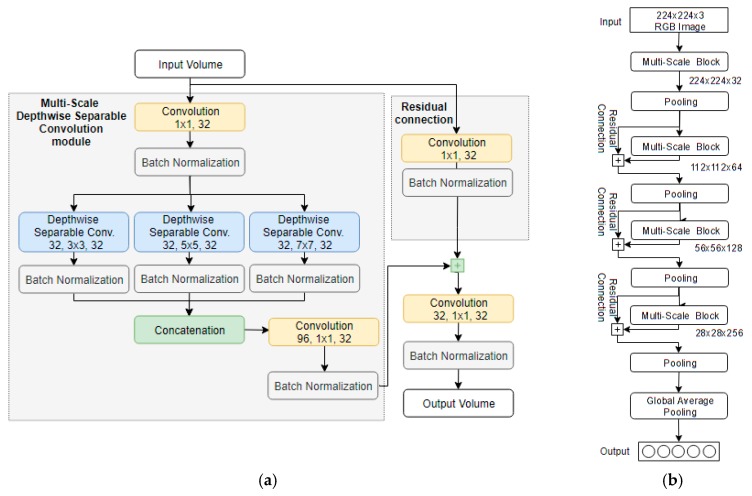
Visualization of (**a**) the multi-scale depthwise separable convolution block and (**b**) the overall Look Behind Fully Convolutional Network (LB-FCN) light network architecture.

For the object recognition task, the LB-FCN light network architecture [45] was chosen, since it has been proven to work well on obstacle detection-related tasks. A key characteristic of the architecture is the relatively low number of free-parameters compared to both conventional CNN architectures, such as [40], and mobile-oriented architectures, such as [41,42]. The LB-FCN light architecture uses Multi-Scale Depth-wise Separable Convolution modules (Figure 12a) to extract features under three different scales, 3 × 3, 5 × 5, and 7 × 7, which are then concatenated, forming a feature-rich representation of the input volume. Instead of conventional convolution layers, the architecture uses depth-wise separable convolutions [43], which drastically reduce the number of free-parameters in the network. 

The combination of the multi-scale modules and depth-wise separable convolutions enables the reduction of the overall computational complexity of the model without sacrificing significant classification performance. Furthermore, the network uses shortcut connections that connect the input with the output of each multi-scale module, promoting the high-level features to be propagated across the network and encounter the problem of vanishing gradient, which is typical in deep networks. Following the principles established in [56], the architecture is fully convolutional, which simplifies the overall network design and lowers further the number of free-parameters. Throughout the architecture, all convolution layers use ReLU activations and more specifically the capped ReLU activation proposed in [41]. As a regularization technique, batch normalization [57] is applied on the output of each convolution layer, enabling the network to converge faster while reducing the incidence of the overfitting phenomenon during training. It is important to note that compared to the conventional CNN architectures used by other VCP assistance frameworks, such as [21,28,29], the LB-FCN light architecture offers significantly lower computational complexity with high classification accuracy, making it a better choice for the proposed system. 

## 5. Experimental Framework and Results

To validate the proposed system, a new dataset was constructed consisting of videos captured from an area of cultural interest, namely the Ancient Agora of Athens, Greece. The videos were captured using a RealSense D435 mounted on the smart glasses (Section 3.2) and were divided into two categories. The first category focused on videos of free walk around the area of Ancient Agora and the second category on controlled trajectories towards obstacles found in the same area.

The validation of the system was developed around both obstacle detection and their class recognition. When an obstacle was identified and its boundaries were determined, the area of the obstacle was cropped and propagated to the obstacle recognition network. In the rest of this section, the experimental framework will be further described (Section 5.1) along with results achieved using the proposed methodology (Section 5.2).

### 5.1. Experimental Framework

The dataset composed for the purposes of this study focuses on vertical obstacles that can be found in sites of cultural interest. The dataset consisted of 15,415 video frames captured by researchers wearing the smart glasses described Section 3.2 (Figure 2). In 5138 video frames the person wearing the camera was walking towards the obstacles but not in a range for the obstacle to be considered threatening. In the rest 10,277 video frames, the person was walking until collision, towards obstacles considered as threatening, which should be detected and recognized. The intervals determining whether an obstacle is considered as threatening or not were set according to the user requirements established by VCP for obstacle detection tasks in [17]. Regarding that, the desired detection distance for the early avoidance of an obstacle according to the VCP user requirements is up to 2 m.

**Figure 13:**
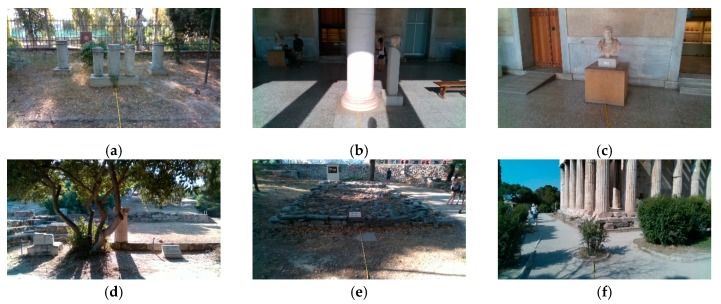
Example of the objects identified as obstacles in our dataset: (**a**–**c**) columns/artifacts; (**d**) tree; (**e**) cultural sight near the ground level; (**f**) small tree/bush.

During data collection, the camera captured RGB images, corresponding depth maps, and stereo infrared (IR) images. The D435 sensor is equipped with an IR projector, which is used for the improvement of depth quality through the projection of an IR pattern that enables texture enrichment. The IR projector was used during the data acquisition for a more accurate estimation of the depth. In this study, only the RGB images and the depth maps needed for our methodology were used. The categories of obstacles visible in the dataset were columns, trees, archaeological artifacts, crowds, and stones. An example of types of obstacles included in our dataset can be seen in Figure 13. As previously mentioned, all data were captured in an outdoor environment, in the Ancient Agora of Athens. In addition, it is worth noting that the data collection protocol that was followed excludes any images that include human subjects that could be recognized in any way. 

### 5.2. Obstacle Detection Results

For the obstacle detection task, only the high-risk map was used, since it depicts objects that pose immediate threat to the VCP navigating the area. The high-risk interval of the membership function *r*_1_ was decided to be at 0 < *z* < 3.5 m. By utilizing the fuzzy sets, an immediate threat within the range of 0 < *z* < 1.5 m can be identified, since the responses of *r*_1_ in this interval are 1, and then, it degrades until the distance of 3.5 m, where it becomes 0. With this approach, the uncertainty within the interval of 1.5 < *z* < 3.5 m is taken into consideration, while at the same time, the requirement regarding the detection up to 2 m is satisfied. The GAN that was used for the estimation of the saliency maps based on the human eye-fixation was trained on the SALICON dataset [58].

The proposed methodology was evaluated on the dataset described in Section 4.1. For the evaluation of the obstacle detection methodology, the sensitivity, specificity, and accuracy metrics were used. The sensitivity and specificity are formally defined as follows:

(14) $$Sensitivity=\frac{TP}{TP+FN}$$

(15) $$Specificity=\frac{TN}{TN+FP}$$

where *TP* (true positive) are the true positive obstacle detections, e.g., the obstacles that were correctly detected, *FP* (false positive) are the falsely detected obstacles, *TN* (true negative) are frames were correctly no obstacles were detected, and *FN* (false negative) are frames that obstacles were not correctly detected. 

**Figure 14:**
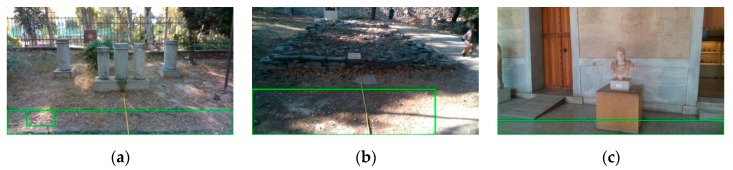
Qualitative example of false ground detection as obstacle resulting from using the methodology presented in [38]. In all images, the obstacles are not in a threatening distance. (**a**) False positive detection on dirt ground-type. (**b**) False positive detection on rough dirt ground-type. (**c**) False positive detection on tile ground-type.

Our method resulted in an accuracy of 85.7% on its application of the aforementioned dataset, with a sensitivity and specificity of 85.9% and 85.2%, respectively. A confusion matrix for the proposed method is presented in Table 1. For further evaluation, the proposed method was compared to that proposed in [38], which, on the same dataset, resulted in an accuracy of 72.6% with a sensitivity and specificity of 91.7% and 38.6%, respectively. The method proposed in [38] included neither the ground plane removal in its pipeline nor the personalization aspect. On the other hand, the proposed approach was greatly benefited from these aspects in the minimization of false alarms. As it can be seen in Figure 14, the dataset contains frames where the camera is oriented towards the ground, and without a ground plane removal step, false alarms are inevitable. The obstacles in Figure 14 were not in a range to be identified as a threat to the user; however, in Figure 14a–c, where the ground plane removal has not been applied, the ground has been falsely identified (green boxes) as obstacle. A quantitative comparison between the two methods can be seen in Table 2.

**Figure 15:**
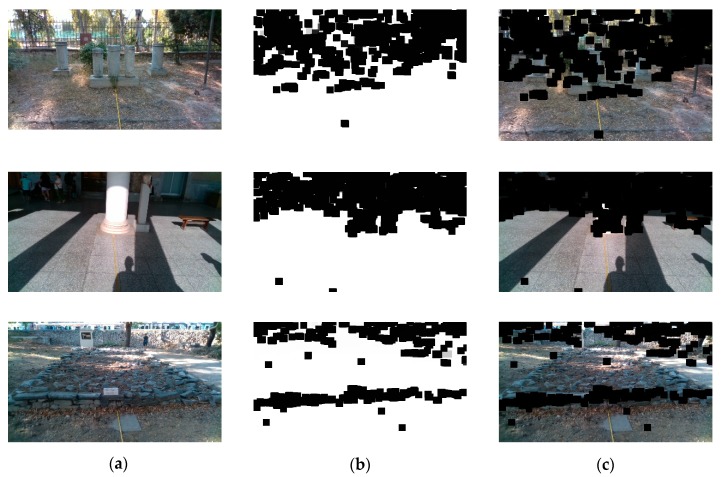
Qualitative representation of the ground removal method. (**a**) Original *I_RGB_* images. (**b**) Ground masks with the white areas indicating the ground plane. (**c**) Images of (**a**) masked with the masks of (**b**).

Qualitative results with respect to the ground detection method can be seen in Figure 15. As it can be observed, the methodology used for the ground plane detection is resilient to different ground types. The ground types that were found in our dataset were grounds with dirt, tiles, marble, and gravels. In addition, using such a method reduces greatly the false alarm rate when the head is oriented towards the ground plane. Even though the masking process is noisy, the obstacle inference procedure is not affected. 

### 5.3. Obstacle Recognition Results

**Figure 16:**
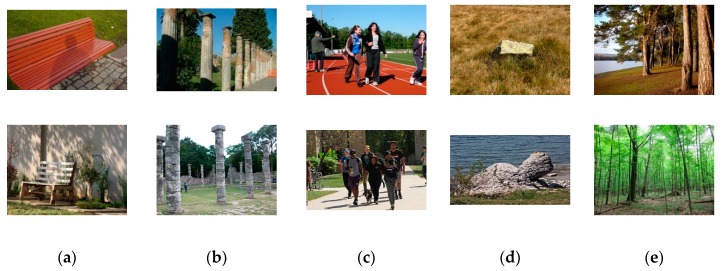
Sample images from the five obstacle categories: (**a**) “benches”, (**b**) “columns”, (**c**) “crowd”, (**d**) “stones”, and (**e**) “trees” from the “Flickr Obstacle Recognition” dataset.

The original LB-FCN light architecture was trained on the binary classification problem of staircase detection in outdoor environments. In order to train the network on obstacles that can be found by the VPS, a new dataset named “Flickr Obstacle Recognition” was created (Figure 16) with images, published under the Creative Commons license, found on the popular social media platform “Flickr” [59]. The dataset contains 1646 RGB images of various sizes that contain common obstacles, which can be found in the open space. More specifically, the images are weakly annotated based on their content in 5 obstacle categories: “benches” (427 images), “columns” (229 images), “crowd” (265 images), “stones” (224 images), and “trees” (501 images). It is worth mentioning that the dataset is considered relatively challenging, since the images were obtained by different modalities, under various lighting conditions and different landscapes. 

For the implementation of the LB-FCN light architecture, the popular Keras [60] python library with the Tensorflow [61] was used as the backend tensor graph framework. To train the network, the images were downscaled to a size of 224 × 224 pixels and zero-padded where needed to maintain the original aspect ratio. No further pre-processing was applied to the images. For the network training, the Adam [62] optimizer was used with an initial learning rate of alpha = 0.001 and first and second moment estimates exponential decay as rate beta1 = 0.9 and beta2 = 0.999, respectively. The network was trained using a high-end NVIDIA 1080TI GPU equipped with 3584 CUDA cores [63], 11 GB of GDDR5X RAM, and base clock speed of 1480 MHz. 

To evaluate the recognition performance of the trained model, the testing images were composed by the detected objects found by the object detection component of the system. More specifically, 212 obstacles of various sizes were detected. The pre-processing of the validation images was similar to that described above for the training set.

For comparison, the state-of-the-art mobile-oriented architecture named “MobileNet-v2” [64] was trained and tested using the same training and testing data. The comparative results, presented in Table 3, demonstrate that the LB-FCN light architecture is able to achieve higher recognition performance, while requiring lower computational complexity, compared to the MobileNet-v2 architecture (Table 4). 

## 6. Discussion

Current imaging, computer vision, speech, and decision-making technologies have the potential to further evolve and be incorporated into effective assistive systems for the navigation and guidance of VCPs. The present study explored novel solutions to the identified challenges, with the aim to deliver an integrated system with enhanced usability and accessibility. Key features in the context of such a system are obstacle detection, recognition, easily interpretable feedback for the effective obstacle avoidance, and a novel system architecture. Some obstacle detection methods such as [21] tackle the problem by incorporating deep learning methods for the obstacle detection tasks and using only the 2D traits of the images. In this work, a novel method was presented, where the 3D information acquired using an RGB-D sensor was exploited for the risk assessment from the depth values of the scenery using fuzzy sets. The human eye fixation was also taken into consideration, estimated by a GAN, in terms of saliency maps. The fuzzy aggregation of the risk estimates and the human eye fixation had as a result the efficient detection of obstacles in the scenery. In contrast to other depth-aware methods, such as the one proposed in [36], the obstacles detected with our approach are described with linguistic values with regard to their opposing risk and spatial location, making them easily interpretable by the VCP. In addition, the proposed method does not only extract obstacles that are an immediate threat to the VCP, e.g., these with non-zero responses from the high-risk membership function *r*_1_, but also obstacles that are of medium and low risk. Therefore, all obstacles are known at any time, even if they are not of immediate high risk. The personalization aspects of the proposed method, alongside with the ground plane detection and removal, provide a significant lower false alarm rate. Furthermore, the method is able to detect and notify the user about partially visible obstacles with the condition that the part of the obstacle is: (a) salient, (b) within a distance that would be considered of high risk and (c) at a height that would be affecting the user. In detail, the overall accuracy of the system based on the proposed method was estimated to be 85.7%, when the methodology proposed in [38] produced an accuracy of 72.6%, based on the dataset described in Section 4.1. Additionally, in contrast to other methodologies such as [2,26,27,31,32], the proposed obstacle detection and recognition system is solely based on visual cues obtained using only an RGB-D sensor, minimizing the computational and energy resources required for the integration, fusion, and synchronization of multiple sensors.

Over the years, there has been a lot of work in the field of deep learning that tempts to increase the classification performance in object recognition tasks. Networks, such as VGGNet [40], GoogLeNet [65], and ResNet [66] provide high classification accuracy but with ever more increasing computational complexity, the result of which limits their usage on high-end devices equipped with expensive GPUs and low inference time [67]. Aiming to decrease the computational complexity and maintain high object recognition performance, this work demonstrated that the LB-FCN light [45] architecture can be used as an effective object recognition solution in the field of obstacle recognition. Furthermore, the comparative results presented in Section 5.2 exhibited that the LB-FCN light architecture is able to achieve higher generalization performance and maintain lower computational complexity compared to the state-of-the-art MobileNet-v2 architecture [64]. It is worth mentioning that single shot detectors, such as YOLO [34] and its variances, have been proved effective in object detection and recognition tasks. However, such detectors are fully supervised, and they need to be trained on a dataset with specific kinds of objects to be able to recognize them. In the current VPS, the obstacle detection task is handled by the described fuzzy-based methodology, which does not require any training on domain-specific data. Therefore, its obstacle detection capabilities are not limited by previous knowledge about the obstacles, and in that sense, it can be considered as a safer option for the VCPs. Using LB-FCN light, which is fully supervised, on top of the results of the fuzzy-based obstacle detection methodology, the system is able to recognize obstacles of predefined categories, without jeopardizing the user’s safety. Although the trained model achieved a high overall object recognition accuracy of 93.8%, we believe that by increasing the diversity of the training “Flickr Object Recognition” dataset, the network can achieve an even higher classification performance. This is due to the fact that the original training dataset contains obstacles located in places and terrains that differ a lot from the ones found in the testing dataset.

The human-centered system architecture presented in Section 3.1 orchestrates all the different components of the VPS. The combination of the BCU component with the RGB-D stereoscopic camera and a Bluetooth headset, all mounted on a 3D printed wearable glass frame, enables the user to move freely around the scenery without attracting unwelcome attention. Furthermore, the cloud computing component of the architecture, enables transparent horizontal infrastructure scaling, allowing the system to be expanded based on future needs. Lastly, the communication protocols used by the different components of the system enable transparent component replacement without requiring any redesign of the proposed architecture.

In order to address and integrate the user and design requirements in the different stages of system development, the design process needs to be human-centered. The user requirements for assistive systems, focused on the guidance of VCP, have been extensively reviewed in [17]. Most of the requirements concerned audio-based functions; tactile functions; functions for guidance and description of the surrounding environment; connectivity issues; and design-oriented requirements such as battery life, device size, and device appearance. Relevant wearable systems have embodied, among others, battery and controller [14], 3D cameras with large on-board FPGA processors [68], and inelegant frame design [16], which are contrary to certain user requirements concerning size/weight, aesthetics, and complexity, described in [17]. A major advantage of the proposed configuration is its simplicity, since it includes only the camera and one cable connected to a mobile device. On the contrary, a limitation of the current system is the weight of the camera, which may cause discomfort to the user. Most of this weight is due to the aluminum case. A solution to this issue is to replace the camera with its caseless version, which is commercially available, and make proper adjustments to the designed frame. 

## 7. Conclusions

In this work, we presented a novel methodology to tackle the problem of visually challenged mobility assistance by creating a system that implements:

- A novel uncertainty-aware obstacle detection methodology, exploiting the human eye-fixation saliency estimation and person-specific characteristics;
- Integration of obstacle detection and recognition methodologies in a unified manner;
- A novel system architecture that allows horizontal resource scaling and processing module interchange ability.

More specifically, the proposed VPS incorporates a stereoscopic camera mounted on an adjustable wearable frame, providing efficient real-time personalized object detection and recognition capabilities. Linguistic values can describe the position and type of the detected object, enabling the system to provide an almost natural interpretation of the environment. The 3D printed model of the wearable glasses was designed based on the RealSense D435 camera, providing a discreet and unobtrusive wearable system that should not attract undue or unwelcome attention. 

The novel approach followed by the object detection module employs fuzzy sets along with human eye fixation prediction using GANs and enables the system to perform efficient real-time object detection with high accuracy prevailing in current state-of-the-art approaches. This is achieved by incorporating depth-maps along with saliency maps. The module is capable to accurately locate an object that poses a threat to the person navigating the scenery. For the object recognition task, the proposed system incorporates deep learning to recognize the objects obtained from the object detection module. More specifically, we use the state-of-the-art object recognition CNN, named LB-FCN light, which offers high recognition accuracy with relatively low number of free parameters. To train the network, a new dataset was created, named “Flickr Obstacle Recognition” dataset, containing RGB outdoor images from five common obstacle categories. 

The novel object detection and recognition modules, combined with the user-friendly and highly adjusTable 3D frame, suggest that the proposed system can be the backbone for the development of a complete, flexible, and effective solution to the problem of visually challenged navigation assistance. The effectiveness of the proposed system was validated for both obstacle detection and recognition using datasets acquired from an outdoor area of interest. As a future work we intend to further validate our system in field tests where VCPs and/or blind-folded subjects will wear the proposed VPS for outdoor navigation. the capacity for further improvements of the background algorithms, structural design, and incorporated equipment provides great potential to the production of a fully autonomous commercial product, available to everyone at low cost. Furthermore, considering that the proposed VPS is developed in the context of a project for assisted navigation in cultural environments, the acquired data can be used also for the 4D reconstruction of places of cultural importance, by exploiting and improving state-of-the-art approaches [69,70]. Such a functionality extension of the system will contribute to further enhancement of cultural experiences for a broader userbase, beyond VCPs, as well as to the creation of digital archives with research material for the investigation of cultural environments over time, via immersive 4D models.

## Figures and Tables

**Table 1:** Confusion matrix of the proposed methodology. Positive are the frames with obstacles, and negative are the frames with no obstacles.

	Detected	
Actual	Positive (%)	Negative (%)
Positive (%)	55.1	9.0
Negative (%)	5.3	30.6

**Table 2:** Results and quantitative comparison between the proposed and state-of-the art methodologies.

Metrics	Proposed (%)	Method [38] (%)	Method [36] (%)
Accuracy	85.7	72.6	63.7
Sensitivity	86.0	91.7	87.3
Specificity	85.2	38.6	21.6

**Table 3:** Comparative classification performance results between the LB-FCN light architecture [45] and the MobileNet-v2 architecture [64].

Metrics	LB-FCN Light [45] (%)	MobileNet-v2 [64] (%)
Accuracy	93.8	91.4
Sensitivity	92.4	90.5
Specificity	91.3	91.1

**Table 4:** Computational complexity comparison between the LB-FCN light architecture [3] and the MobileNet-v2 architecture [65].

Metrics	LB-FCN Light [45] (%)	MobileNet-v2 [64] (%)
FLOPs × $10^{6}$	0.6	4.7
Trainable free parameters × $10^{6}$	0.3	2.2
